# Supplemental *Bacillus subtilis* PB6 Improves Growth Performance and Gut Health in Broilers Challenged with *Clostridium perfringens*

**DOI:** 10.1155/2021/2549541

**Published:** 2021-10-27

**Authors:** Yan Liu, Song Zhang, Zheng Luo, Dan Liu

**Affiliations:** ^1^State Key Laboratory of Animal Nutrition, College of Animal Science and Technology, China Agricultural University, Beijing, China; ^2^Department of Animal Resource & Science, Dankook University, Cheonan, Choongnam 330–714, Republic of Korea; ^3^Kemin (China) Technologies Co. Ltd., 25 Qinshi Road, Sanzao, Zhuhai 519040, China

## Abstract

*Clostridium perfringens* (CP) is the principal pathogenic bacterium of chicken necrotic enteritis (NE), which causes substantial economic losses in poultry worldwide. Although probiotics are known to provide multiple benefits, little is known about the potential effects of *Bacillus subtilis* (*B*. *subtilis*) application in preventing CP-induced necrotic enteritis. In this study, 450 male Arbor Acres broilers were divided into 5 experimental treatments: A: basal diet (control group); B: basal diet and CP challenge (model group); C: CP challenge+10 mg/kg enramycin (positive control group); D: CP challenge+4 × 10^7^ CFU/kg of feed *B*. *subtilis* PB6 (PB6 low-dosage group); and E: CP challenge+6 × 10^7^ CFU/kg of feed *B*. *subtilis* PB6 (PB6 high-dosage group). There were 6 replicate pens per treatment with 15 broilers per pen. The present research examined the effect of *Bacillus subtilis* PB6 (*B*. *subtilis* PB6) on growth performance, mRNA expression of intestinal cytokines and tight junctions, and gut flora composition in broilers challenged with CP. The entire experiment was divided into two phases: the non-CP challenge phase (d0–18) and the CP challenge phase (d18–26). PB6 did not increase the growth performance during the first stage, but the PB6 high-dosage group was found to have larger body weight gain and ADFI during the CP challenge stage. Feed supplementation with PB6 reduced the lesion score of challenged chicks, with increased tight junction-related gene expression (*occludin* and *ZO*-*1*) and decreased *TNF*-*α* expression compared with CP-infected birds. A decrease in the abundance of *Clostridium XI*, *Streptococcu*s, and *Staphylococcus* was observed after CP infection (*P* < 0.05), while supplementation with PB6 restored the ileal microbial composition. In conclusion, administration of *B*. *subtilis* PB6 improved growth performance, enhanced intestinal barrier function, and mitigated intestinal inflammation/lesions, which might be due to its restoring effects on the ileal microbial composition in CP-challenged broilers.

## 1. Introduction

Necrotic enteritis (NE) is a serious bacterial disease in poultry that causes devastating financial losses. *Clostridium perfringens* (CP), an anaerobic gram-positive bacterium, is the major pathogen of NE. In fact, CP is one of the gut symbiotic bacteria of birds, and healthy birds normally harbor 10^4^ CFU of CP/g of digesta in their intestine. CP is an opportunistic pathogen in some conditions, such as dietary nutrient risks (unbalanced diet formulation, antinutritional factors, and quality of raw materials), disease challenges (coccidiosis, infectious bursal virus), and inadequate management (high temperature), with increased proliferation to ~10^7^ to 10^9^ CFU/g of digesta [1], which leads to clinical signs of NE. In poultry production, subclinical NE leads to significant economic losses due to lesions in the small intestine, which in turn reduces body weight gain (BWG) and impairs the feed conversion ratio (FCR) [2]. Chickens are a reservoir of CP, and contaminated chicken products also represent a potential public food safety threat [3]. In human medicine, *Clostridial*-contaminated food can be treated with surgical debridement and oral antibiotic therapy. However, antibiotic resistance among anaerobic bacteria, such as *Clostridial* species, is increasing worldwide [4]. Therefore, an efficient and natural method to overcome NE for chicken production in the postantibiotic age is warranted.

Probiotic supplementation has been demonstrated to be an efficient natural approach for regulating intestinal flora in humans and farm animals, which can act as follows: (a) a sustainer of the intestinal microflora ecosystem to maintain beneficial microflora colonization and inhibit pathogen proliferation, (b) a digestive booster to increase endogenous digestive enzyme activities and indigestion and reduce indigestible nutrient fermentation by depressing the activity of bacterial enzymes, and (c) a positive immune modulator by maintaining intestinal integrity [5]. Meanwhile, qualified probiotics have the capacity to overcome erratic elements, such as gastric acids, bile acids, endogenous proteases, and competition with other microorganisms. Consequently, *Bacillus subtilis* (*B*. *subtilis*) is a widely adopted probiotic bacterial species with many advantages. As a spore-forming facultative anaerobe, it has strong heat resistance and can last for 8 minutes at a high temperature of 113°C, which increases its possibility of survival during feed processing. At the same time, *Bacillus* spores have strong stress resistance and can survive in the gastrointestinal environment under harsh conditions, such as low pH and bile salts [6]. As a symbiotic bacterium, *B*. *subtilis* PB6 (ATCC-PTA 6737) has been proven to produce antibacterial substances and has a wide range of activities against numerous strains *in vitro*, including *Campylobacter* spp. and *Clostridium* spp. [7]. Moreover, research has demonstrated that supplementation with *B*. *subtilis* PB6 alleviates CP-induced gut lesions and also strengthens intestinal barrier function in broilers [8]. However, the potential effects of *B*. *subtilis* PB6 administration on gut microbiota composition and intestinal inflammatory damage in CP-challenged birds remain elusive. Here, we sought to determine the influence of *B*. *subtilis* PB6 administration on growth performance, lesion scores, intestinal tight junctions (TJs), proinflammatory cytokines, and gut microbiota composition in broilers challenged with CP.

## 2. Materials and Methods

### 2.1. Animals, Diets, and Housing

In total, 450 male Arbor Acres broilers were separated into 5 experimental groups, each of which was replicated 6 times for 15 broilers per replicate. The experiments were ethically approved by the Animal Care and Use Committee of China Agricultural University. All treatments were as follows: A: basal diet; B: basal diet+CP challenge; C: CP challenge+10 mg/kg enramycin; D: CP challenge+4 × 10^7^ CFU/kg of *B*. *subtilis* PB6; and E: CP challenge+6 × 10^7^ CFU/kg of *B*. *subtilis* PB6. The original strain of *B*. *subtilis* PB6 was obtained from Kemin China Technologies Co., Ltd., Zhuhai, China. Broilers were raised in a controlled environment and allowed *ad libitum* access to water and feed. Repetitions of different treatments were equally distributed among the cages as much as possible to reduce variations at the cage level. The entire experiment was divided into two phases: the non-CP challenge phase (d0–18) and the CP challenge phase (d18–26).

All diets were designed following the instructions of NRC (1994) and the Chinese chicken feeding standard (NY/T-33–2004) (Table 1). The CP challenge was performed on the basis of the study of Liu et al. [9].

A field strain of CP type A (CVCC2030) was cultured on tryptone-sulfite-cycloserine agar, and a single colony was then inoculated into a cooked meat medium and subsequently cultured in an incubator at 37°C for 8 hours. In the infected groups, chickens were orally inoculated with 1 ml bacterial solution containing 10^8^ CFU/ml CP once a day from Day 19 to Day 25. Chickens in the basal diet group were subjected to the same gavage procedure described above but with a sterilized medium.

### 2.2. Growth Performance

The mortality rate was recorded throughout the experiment. Body weights of chickens were measured on Day 0, on Day 18, and on the last day (Day 26). Feed intake (FI), body weight gain (BWG), and the feed conversion ratio (FCR) were calculated and recorded for further analysis.

### 2.3. Lesion Score

Intestinal scoring was evaluated on Day 26. A randomly selected chicken of each repetition was killed by cervical dislocation and then underwent intestinal scoring. The intestines were cut open and scored for NE lesions. Specifically, after intestinal observation, the intestinal lesions were rated from 0 to 4 points according to the method of Dahiya et al. [10].

### 2.4. Proinflammatory Cytokine and Tight Junction Protein Gene Expression

Jejunum samples were collected from broilers that were randomly selected from each replicate on Day 26 and stored at -80°C for RNA extraction. RNA extraction, reverse transcription, and quantification methods were performed according to Wang et al. [11]. In short, total RNA extraction was performed with a TRIzol reagent, and RNA quality and concentration were detected by a NanoDrop spectrophotometer (ND-2000 UV-Vis; Thermo Scientific Inc.). RNA reverse transcription and real-time fluorescence quantification were carried out with Takara reagents following the manufacturer's instructions (Takara Biotechnology Inc.). An ABI 7500 Real-Time PCR System (Applied Biosystems) was used for real-time fluorescence quantitative detection. *β*-Actin was used as a reference gene to normalize the relative RNA expression. The primer sequences for *β*-actin, occludin, ZO-1, TLR4, IL-1*β*, TNF-*α*, and IFN-*γ* are listed in Table 2. Each sample was measured in triplicate, and the average value was calculated. The 2^-*ΔΔ*^Ct method was used to calculate the relative mRNA expression of target genes [12].

### 2.5. Pyrosequencing of Ileal Microbiota

On Day 26, broiler ileal digesta were sampled. DNA was extracted from ileal digesta using a QIAamp® Fast DNA Stool Mini Kit (Qiagen Ltd., Germany) according to the guidelines. According to the specifications outlined by Illumina, all DNA samples were pretreated for MiSeq compositional sequencing. The V3-V4 region of the 16S rRNA gene was amplified, and Illumina index primers were attached in two separate PCRs.

FLASH software (v1.2.7) was used to generate raw tags [13]. Effective tags were obtained by the UCHIME algorithm [14] and QIIME (v1.7.0) analysis [15]. UPARSE software (v7.0.1001) was used to analyze sequences, and the sequences were clustered at 97% similarity as operational taxa (OTUs). The GreenGene database was used to compare sequences and classify the different classification levels of these sequences. Microbial diversity was detected through QIIME software and Python scripts.

### 2.6. Statistical Analysis

The data, including growth performance, lesion scores, and the gene expression of both intestinal tight junctions and proinflammatory cytokines collected for quantitative parameters, were analyzed using analysis of variance (ANOVA) under a completely randomized design. Significant differences among the treatments were measured using Duncan's multiple comparison and were declared when *P* < 0.05.

## 3. Results

### 3.1. Effects of *Bacillus subtilis* PB6 on Growth Performance in Broilers Challenged with *Clostridium perfringens*

As shown in Table 3, before the birds were challenged by CP (Days 1–18), no differences were detected among the treatment groups (*P* > 0.05). However, during the CP challenge period, CP infection significantly reduced BWG and ADFI (*P* < 0.05) and tended to increase FCR (*P* = 0.062) compared to the control group. However, the addition of enramycin numerically increased the BWG of CP-infected chicks (*P* > 0.05). Moreover, the addition of a low dosage of *B*. *subtilis* PB6 increased the BWG (*P* < 0.05) and tended to increase the ADFI (*P* > 0.05) of CP-challenged chicks, while the addition of a high dosage of *B*. *subtilis* PB6 significantly increased the BWG and ADIF of CP-challenged chicks (*P* < 0.05).

### 3.2. Effects of *Bacillus subtilis* PB6 on Intestinal Lesions and Gene Expression in Broilers Challenged with *Clostridium perfringens*

As shown in Table 4, CP infection significantly increased the intestinal lesion score compared to the control group (*P* < 0.05). However, dietary addition of enramycin and low and high dosages of *B*. *subtilis* PB6 significantly reduced the intestinal lesion score of CP-infected broilers (*P* < 0.05).

Compared to the control group, CP challenge led to a significant decrease in jejunum mRNA expression of occludin (*P* < 0.05) and tended to upregulate TNF-*α* expression (*P* > 0.05). Nevertheless, supplementation with enramycin upregulated occludin expression and downregulated TNF-*α* expression in the jejunum of CP-infected birds (*P* < 0.05). Moreover, the infected broilers fed a diet with a low dosage of *B*. *subtilis* PB6 showed the highest ZO-1 mRNA expression among all groups (*P* < 0.05) and had relatively higher occludin expression than CP-infected birds (*P* > 0.05). Infected birds fed a diet with a high dosage of *B*. *subtilis* PB6 had lower expression of TNF-*α* (*P* < 0.05) and relatively higher occludin expression (*P* > 0.05) than infected birds fed a basal diet.

### 3.3. The Quality of Gut Microbiota Sequencing Data

**Figure 1:**
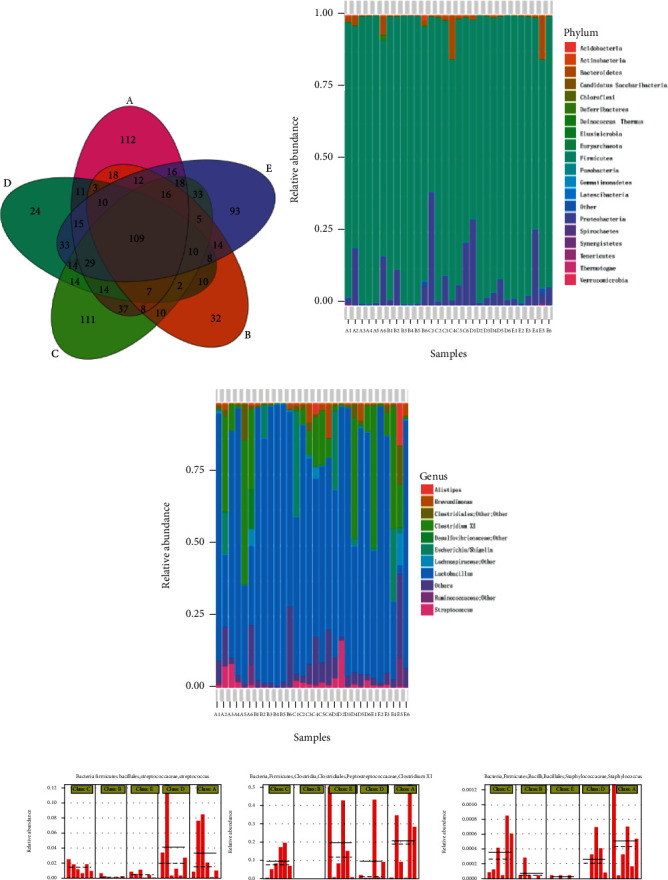
Effects of *Bacillus subtilis* PB6 on the intestinal bacterial structure in broilers challenged with *Clostridium perfringens*. (a) Venn diagrams showing the shared OTUs between the five different treatments. (b) Effects of relative abundance (%) of ileal bacterial taxa at the phylum level of broilers. (c) Relative abundance (%) of ileal bacterial taxa at the genus level of broilers. (d) The relative abundance (%) of ileal bacterial taxa at the genus level of *Streptococcus*, *Clostridium XI*, and *Staphylococcus* in broilers. Treatment information: A: basal diet; B: basal diet and CP challenge; C: CP challenge+10 mg/kg enramycin (positive control group); D: CP challenge+4 × 10^7^ CFU/kg of feed *B*. *subtilis* PB6 (PB6 low-dosage group); E: CP challenge+6 × 10^7^ CFU/kg of feed *B*. *subtilis* PB6 (PB6 high-dosage group).

After the OTUs were assigned and chimeras were removed, 1,965,036 effective sequences were obtained from 30 ileal samples, and 56,143 sequences were shared by a single sample. The read length ranged from 220 to 500 base pairs (bp), and the median read length was 427 bp. OTU numbers were identified. As shown in Figure 1(a), 109 OTUs were shared by five groups, and 112, 32, 111, 24, and 93 OTUs were exclusive in each group.

### 3.4. Effects of *Bacillus subtilis* PB6 on the Intestinal Bacterial Structure and Diversity in Broilers Challenged with *Clostridium perfringens*

Phylum-level microbiota analysis showed that *Firmicutes* (89.97%), *Proteobacteria* (7.67%), and *Bacteroidetes* (1.78%) (Figure 1(b)) were the three most dominant phyla, accounting for 99.42% of all sequences in the groups. Supplementation with enramycin reduced *Firmicutes* abundance in the ileum. As shown in Figure 1(c), the analysis of the ileal community at the genus level revealed that *Lactobacillus* (69.63%), *Clostridium XI* (11.70%), *Escherichia* (4.24%), *Streptococcus* (1.92%), and *Brevundimonas* (1.44%) were the five dominant genera.

**Figure 2:**
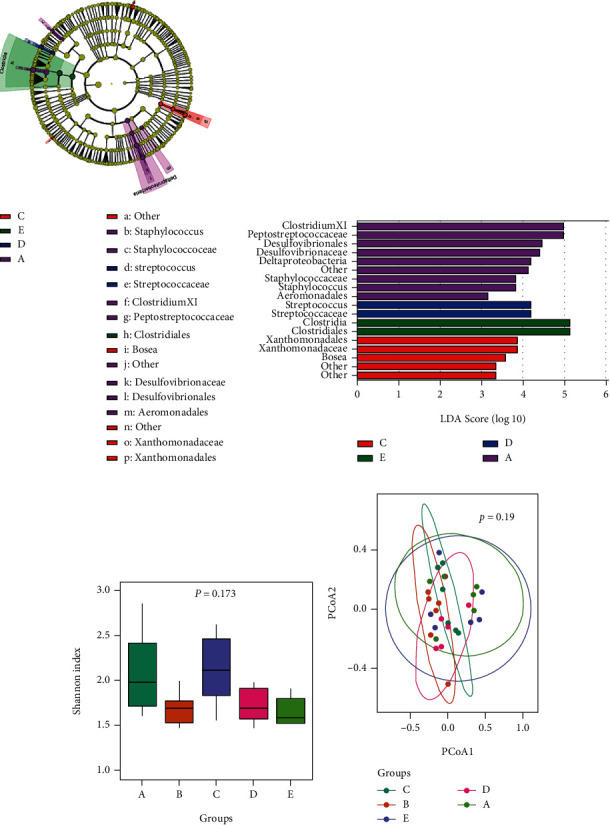
Effects of *Bacillus subtilis* PB6 on intestinal bacterial diversity in broilers challenged with *Clostridium perfringens*. (a) Diversity and composition of ileal microbiota (LEfSe score) in broilers. (b) Diversity and composition of ileal microbiota (circular cladogram) in broilers. (c) Alpha diversity analysis (Shannon) of ileal microbiota in broilers. (d) Beta diversity analysis (PCoA) of ileal microbiota in broilers. Treatment information: A: basal diet; B: basal diet and CP challenge; C: CP challenge+10 mg/kg enramycin (positive control group); D: CP challenge+4 × 10^7^ CFU/kg of feed *B*. *subtilis* PB6 (PB6 low-dosage group); E: CP challenge+6 × 10^7^ CFU/kg of feed *B*. *subtilis* PB6 (PB6 high-dosage group).

Further statistics were carried out to identify differences in the ileal community at the genus level among the groups, which are shown in Figure 1(d). CP challenge significantly decreased the abundance of *Clostridium XI*, *Streptococcu*s, and *Staphylococcus* in comparison with the control group (*P* < 0.05), while supplementation with enramycin and low-dosage *B*. *subtilis* PB6 raised these bacterial abundances to levels similar to those of nonchallenge birds (*P* > 0.05). The birds fed high-dosage *B*. *subtilis* PB6 had more *Clostridium XI* than the other groups (*P* < 0.05). LEfSe analysis showed that 18 differentially abundant bacterial clades were distributed to all taxonomic levels (LDA score > 3.0) among the 5 treatments (Figure 2(a)). The highest relative abundances of *Clostridium XI*, *Peptostreptococcaceae*, *Desulfovibrionaceae*, and *Staphylococcus* were indicated in the control group among all treatments (LDA score > 3.0). Moreover, supplementation with enramycin increased the relative abundances of *Bosea* and *Xanthomonadaceae* (LDA score > 3.0). Supplementation with a low dosage of *B*. *subtilis* PB6 increased *Streptococcus* abundance (LDA score > 3.0), while supplementation with a high dosage of *B*. *subtilis* PB6 increased *Clostridia* abundance (LDA score > 3.0).  Figure 2(b) shows the ileal microbial alpha diversity. There were no significant differences in the diversity indexes (Shannon) among all groups (*P* > 0.05). Beta diversity was demonstrated via PCoA in Figure 2(c), showing no distinguishable clustering of the ileal samples in different treatments.

## 4. Discussion

Pathogenic bacteria, such as *Clostridium perfringens* (CP), can cause imbalances in animal homeostasis and damage to the body and severely affect animal growth performance [16]. A large amount of evidence has shown that probiotics are beneficial for improving the growth performance of animals under both the pathogen-infected and noninfected conditions, which may be due to the compounds secreted by probiotics, such as digestive enzymes, antibacterial substances, and/or other growth-promoting factors, such as short-chain fatty acids [17, 18]. As a widely used probiotic, *Bacillus subtilis* PB6 has been proven to improve broiler/animal feed intake, increase body weight, and reduce FCR [19, 20]. In the present study, we also found that adding 4 × 10^7^ and 6 × 10^7^ CFU/kg*B*. *subtilis* PB6 to the diet could mitigate the negative effects of CP infection on broiler BWG and FCR, particularly with the addition of a high dose. In addition, we found that *B*. *subtilis* PB6 exerted a better growth-promoting effect on CP-infected broilers than enramycin, which is commonly used in CP prevention, ultimately confirming the growth-promoting effect of *B*. *subtilis* PB6. However, other studies showed that the growth-promoting effect of *B*. *subtilis* PB6 was not significant [21], and these inconsistent results across studies may be due to the breeding environment, animal species, additive dosage, or operating procedures.

CP can produce various toxins, bacteriocins, and collagenolytic enzymes after colonization [22]. These active substances affect tight junctions and their components, such as occludin and junction adhesion molecules (JAMs), by altering the transmembrane pores and extracellular matrix of intestinal cells, leading to compromised integrity of the lamina propria [23]. The above alterations are accompanied by activation of the mucosal immune response; subsequently, many proinflammatory cytokines are secreted, such as TNF-*α* and IL-1*β* [24]. These cytokines can induce the rearrangement of tight junctions and damage the intestinal barrier, thereby causing a vicious cycle over the host and even a systemic infection [22]. In line with other studies [25–27], our results noted that CP infection caused severe physical damage to the intestine of broilers, significantly raised intestinal lesion scores, inhibited the gene expression of the intestinal TJ protein occludin, and upregulated the expression of the proinflammatory cytokine TNF-*α*. These abnormal physiological alterations may be important factors in the reduction of growth performance of broilers infected with CP. Furthermore, we found that enramycin and *B*. *subtilis* PB6 could variously increase the expression of the TJ proteins occludin and ZO-1 in CP-infected broilers and reduce the expression of the inflammatory factor TNF-*α*. This indicates that the addition of enramycin and *B*. *subtilis* PB6 can restore the intestinal physical barrier and reduce intestinal inflammation, thus helping to decrease the intestinal lesion scores and recover the physiological function of the damaged intestine. In agreement with our findings, Jayaraman et al. and Belote et al. reported that supplementation with *B*. *subtilis* PB6 and enramycin prevented CP-induced NE and decreased lesion scores and also improved intestinal health in challenged broilers [8, 28]. Enramycin can kill pathogenic bacteria by directly inhibiting their cell wall formation, thereby reducing the damage caused by pathogens to the body [28]. However, in addition to competitive rejection, probiotics can also exert their growth-promoting effects through immune regulation, secretion of antibacterial molecules, and enhancement of the body's antioxidant capacity. In this study, *B*. *subtilis* PB6 exerted its immunomodulatory effect by downregulating the expression of TNF-*α* in the intestine of CP-infected broilers and simultaneously upregulating the expression of occludin and ZO-1. TNF-*α* is produced by activated monocytes/macrophages and is synthesized in large quantities during the acute phase of bacterial infection, subsequently promoting the activation of downstream immune cells, such as T cells, thus intensifying the inflammatory response and harming the host [29]. Similarly, recent studies have shown that *B*. *subtilis* can reduce intestinal inflammation by modifying the polarization of macrophages, inhibiting the expression of TNF-*α*, and thus protecting the body from bacterial infections [30]. As the main components of epithelial tight junctions, the improved mRNA expression of occludin and ZO-1 can enhance intestinal barrier function and protect intestinal health [31, 32]. Studies have also found that the culture supernatant of *B*. *subtilis* can upregulate the expression of tight junction proteins and mucin 2 in HT-29 cells *in vitro*, but the specific metabolites are not yet known, and further exploration is needed [33].

The intestinal microbial community, which is regulated by many factors, such as food [34], age [35], and additives [36], is very important for the growth and health of broilers due to its ability to promote nutrient digestion and regulate the immune system [37]. In general, the diversity of gut microbiota is closely related to pathogen resistance. The intestinal microbiome can be affected by many factors, such as pathological conditions, antibiotic therapy, dietary supplementation, and housing environment [38]. Recently, Fasina et al. [39] and Li et al. [40] reported that CP infection dramatically reduced the *α*-diversity index of the broiler intestinal microbial community. However, Xu et al. [41] and Zhang et al. [42] indicated the opposite result, which revealed that CP infection significantly increased the *α*-diversity index of the gut microbial community. Moreover, some studies found that CP infection did not affect either the *α*- or *β*-diversity index [43, 44], which is in agreement with the current results. Those authors considered that the discrepancy might be attributed to the following: (a) the different sections of the ileum in which the digesta were collected, (b) diverse CP strains and diet types, and (c) the different durations of CP challenge. Relevant references are limited, and further trials are required.

Studies have shown that the abundance of intestinal *Clostridium XI* is significantly increased, accompanied by decreased gene expression of TNF-*α* and a decreased inflammation index in the colon after treatment with probiotics in a colitis mouse model [45]. Consistently, we found a higher abundance of *Clostridium XI* in the ileum of broilers in the control group, enramycin supplementation group, and low- and high-dosage *B*. *subtilis* PB6 supplementation groups, accompanied by a decrease in TNF-*α* expression and reduced intestinal lesions. Therefore, we speculate that a high abundance of *Clostridium XI* may inhibit intestinal inflammation. LEfSe analysis showed that *Streptococcus* was significantly enriched in the low-dose *B*. *subtilis* PB6 supplementation group. *Streptococcus* contains many probiotic strains, such as *Streptococcus thermophilus*. Recent studies have found that *Streptococcus thermophilus* can increase the expression of the tight junction proteins ZO-1 and ZO-2 in high-fat diet mice and maintain the expression of ZO-1 in a human intestinal epithelial cell line infected with *E*. *coli* [46–48]. Therefore, in this experiment, the increased expression of ZO-1 in the intestine of broilers in the low-dose *B*. *subtilis* PB6 supplementation group may be related to the increased abundance of *Streptococcus*. A previous study showed that *Bosea* contains strains that can secrete a variety of cellulolytic enzymes [49]. In this experiment, enramycin treatment significantly increased the abundance of *Bosea* in the intestine of CP-infected broilers. Thus, the ability of *Bosea* to decompose cellulose and improve the utilization of feed nutrients may be one of the reasons for the increased body weight of broilers in the enramycin group. *Staphylococcus*, as a conditional pathogen, is generally considered harmful to the host, but the reason for its decreased abundance in the ileum of CP-infected broilers remains unclear. This may be due to the occupying effect of CP that inhibits the reproduction of *Staphylococcus*. *Desulfovibrionaceae* and *Xanthomonadaceae* are currently less studied. However, one study found that *Xanthomonadales* is enriched in mice inoculated with Chinese propolis [50], but its function is unclear.

## 5. Conclusion

In summary, administration of *B*. *subtilis* PB6 can improve growth performance by enhancing intestinal barrier function, mitigating intestinal inflammation/lesions, and reshaping the ileal microbial composition in CP-challenged birds.

## Figures and Tables

**Table 1:** Basal diet composition (as-fed basis).

Ingredient (%)	Basal diet
Corn	57.52
Soybean meal (CP > 46%)	36.20
Soy oil	2.14
Limestone	1.13
Dicalcium phosphate	1.97
Salt	0.35
Methionine (99%, DL-form)	0.19
Choline (50%)	0.25
Vitamin premix^1^	0.025
Mineral premix^2^	0.2
Ethoxyquin (66%)	0.03
Total	100.00
Calculated composition^3^ (%)	
Crude protein	21.0
ME (kcal/kg)	2950
Ca	1.00
AP	0.45
Lys	1.11

^1^Provided per kg of diet: vitamin premix (1 kg) contained the following: vitamin A, 50 MIU; vitamin D_3_, 12 MIU; vitamin K_3_, 10 g; vitamin B_1_, 10 g; vitamin B_2_, 32 g; vitamin B_12_, 0.1 g; vitamin E, 0.2 MIU; biotin, 0.5 g; folic acid, 5 g; pantothenic acid, 50 g; niacin, 150 g copper, 4 g; zinc, 90 g; iron, 38 g; manganese, 46.48 g; selenium, 0.1 g; iodine, 0.16 g; cobalt, 0.25 g. ^2^Provided per kg of diet: 150 g copper, 4 g; zinc, 90 g; iron, 38 g; manganese, 46.48 g; selenium, 0.1 g; iodine, 0.16 g; cobalt, 0.25 g. ^3^Calculated value based on the analyzed data for the experimental diets.

**Table 2:** Sequences for real-time PCR primers.

Genes	Primer sequence (5′–3′)	Accession no.
*β*-*Actin*	F: GAGAAATTGTGCGTGACATCA R: CCTGAACCTCTCATTGCC	L08165
*Occludin*	F: ACGGCAGCACCTACCTCAA R: GGGCGAAGAAGCAGATGAG	D21837.1
*ZO*-*1*	F: CTTCAGGTGTTTCTCTTCCTCCTC R: CTGTGGTTTCATGGCTGGATC	XM_413773
*TRL4*	F: GTTCCTGCTGAAATCCCAAA R: TATGGATGTGGCACCTTGAA	NM_001030693
*IL*-*1β*	F: ACTGGGCATCAAGGGCTA R: GGTAGAAGATGAAGCGGGTC	NM_204524
*TNF*-*α*	F: GAGCGTTGACTTGGCTGTC R: AAGCAACAACCAGCTATGCAC	NM_204267
*IFN*-*γ*	F: TAACTCAAGTGGCATAGATGTGGAAG R: GACGCTTATGTTGTTGCTGATGG	NM_008337

^1^Abbreviation: ZO-1: zonula occludens-1; TRL4: Toll-like receptor-4; IL-1*β*: interleukin-1*β*; TNF-*α*: tumor necrosis factor-*α*; IFN-*γ*: interferon-*γ*.

**Table 3:** The effect of *B*. *subtilis* supplementation on growth performance and mortality in CP-challenged broilers.

Treatment^1^	Control group	CP-challenged model group	Antibiotics (positive control group)	*B*. *subtilis* PB6 low-dosage group	*B*. *subtilis* PB6 high-dosage group	SEM^2^	*P* value^3^
d1-d18 nonchallenge phase							
BWG (g)	36.06	35.47	35.44	35.14	35.58	0.25	0.86
FI (g)	51.61	50.87	51.65	51.79	51.97	0.44	0.95
FCR	1.43	1.44	1.46	1.48	1.46	0.01	0.50
Mortality rate	0.00	0.00	0.00	0.00	0.00	0.00	1.00
d19-d26 challenge phase							
BWG (g)	66.12^a^	54.95^c^	59.21^bc^	61.62^ab^	62.86^ab^	1.087	0.009
FI (g)	109.55^a^	99.90^bc^	98.35^c^	106.30^ab^	108.41^a^	1.305	0.007
FCR	1.66	1.83	1.67	1.73	1.73	0.02	0.062
Mortality rate (%)	2.22	3.49	1.11	4.45	3.58	0.907	0.821
Overall							
BWG (g)	46.47^a^	42.21^c^	43.67^bc^	44.31^abc^	45.02^ab^	0.394	0.004
FI (g)	71.66^a^	67.84^b^	67.81^b^	70.66^ab^	71.51^a^	0.544	0.026
FCR	1.55	1.61	1.55	1.6	1.59	0.009	0.151
Mortality rate (%)	2.22	3.49	1.11	4.45	3.58	0.907	0.821

^1^Treatment information: control group: basal diet; CP-challenged model group: basal diet and CP challenge; antibiotics (positive control group): CP challenge+10 mg/kg enramycin; *B*. *subtilis* PB6 low-dosage group: CP challenge+4 × 10^7^ CFU/kg of feed *B*. *subtilis* PB6; *B*. *subtilis* PB6 high-dosage group: CP challenge+6 × 10^7^ CFU/kg of feed *B*. *subtilis* PB6. BWG: body weight gain; FI: feed intake; FCR: feed conversion ratio. ^2^Standard error of the means; *n* = 6 chickens/group. ^3^Mean values within a column with unlike superscripts letters (a, b, and c) are significantly different (*P* < 0.05).

**Table 4:** Intestinal lesion score and relative mRNA expression of intestinal tight junction proteins and proinflammatory cytokines in broilers.

Treatment^1^	Control group	CP-challenged model group	Antibiotics (positive control group)	*B*. *subtilis* PB6 low-dosage group	*B*. *subtilis* PB6 high-dosage group	SEM^2^	*P* value^3^
Occludin	1.11^ab^	0.50^c^	1.39^a^	0.75^bc^	0.65^bc^	0.093	0.009
ZO-1	1.04^c^	1.43^bc^	1.68^abc^	2.54^a^	2.01^ab^	0.145	0.009
TRL4	1.1	1.06	0.98	1.38	1.24	0.069	0.335
IL-1*β*	1.32	2.04	1.47	2.14	2.87	0.199	0.106
TNF-*α*	1.06^ab^	1.45^a^	0.93^b^	1.33^a^	0.95^b^	0.083	0.022
IFN-*γ*	1.12	1.83	1.15	1.68	1.76	0.137	0.289
Lesion score^4^	0.00^d^	1.25^a^	0.33^c^	0.75^b^	0.66^b^	0.088	<0.001

^1^Treatment information: control group: basal diet; CP-challenged model group: basal diet and CP challenge; antibiotics (positive control group): CP challenge+10 mg/kg enramycin; *B*. *subtilis* PB6 low-dosage group: CP challenge+4 × 10^7^ CFU/kg of feed *B*. *subtilis* PB6; *B*. *subtilis* PB6 high-dosage group: CP challenge+6 × 10^7^ CFU/kg of feed *B*. *subtilis* PB6. ZO-1: zonula occludens-1; TRL4: Toll-like receptor-4; IL-1*β*: interleukin-1*β*; TNF-*α*: tumor necrosis factor-*α*; IFN-*γ*: interferon-*γ*. ^2^Standard error of the means; *n* = 6 chickens/group. ^3^Mean values within a column with unlike superscripts letters (a, b, and c) are significantly different (*P* < 0.05). ^4^0 = no gross lesions; 0.5 = severely congested serosa and mesenteric hyperemia; 1 = thin-walled and brittle intestines with small hemorrhagic spots (>5); 2 = small amounts of gas production and focal necrotic lesions; 3 = large amount of gas-filled intestines and necrotic plaques.

## Data Availability

The data used to support the findings of this study are available from the corresponding author upon request.

## Abbreviations

BWG: Body weight gain
ADFI: Average daily feed intake
ZO-1: Zonula occludens-1
TRL4: Toll-like receptor-4
IL-1*β*: Interleukin-1*β*
TNF-*α*: Tumor necrosis factor-*α*
IFN-*γ*: Interferon-*γ*.
